# Cardiac CT and MRI guide surgery in impending left ventricular rupture after acute myocardial infarction

**DOI:** 10.1186/1749-8090-4-42

**Published:** 2009-08-12

**Authors:** Jens Vogel-Claussen, Jan Skrok, Elliot K Fishman, João AC Lima, Ashish S Shah, David A Bluemke

**Affiliations:** 1 Johns Hopkins University School of Medicine, Russell H. Morgan Department of Radiology and Radiological Science, Baltimore, MD, USA; 2 Johns Hopkins University School of Medicine, Department of Cardiology, Baltimore, MD, USA; 3 Johns Hopkins University School of Medicine, Department of Surgery, Division of Cardiac Surgery, Baltimore, MD, USA; 4 National Institutes of Health, Department of Radiology and Imaging Sciences, Bethesda, MD, USA

## Abstract

We report the case of a 67 year-old patient who presented with worsening chest pain and shortness of breath, four days post acute myocardial infarction. Contrast enhanced computed tomography of the chest ruled out a pulmonary embolus but revealed an unexpected small subepicardial aneurysm (SEA) in the lateral left ventricular wall which was confirmed on cardiac magnetic resonance imaging. Intraoperative palpation of the left lateral wall was guided by the cardiac MRI and CT findings and confirmed the presence of focally thinned and weakened myocardium, covered by epicardial fat. An aneurysmorrhaphy was subsequently performed in addition to coronary bypass surgery and a mitral valve repair. The patient was discharged home on post operative day eight in good condition and is feeling well 2 years after surgery.

## Background

The formation of left ventricular (LV) myocardial aneurysms is one of several potentially life-threatening complications post acute myocardial infarct (AMI). These aneurysms are traditionally divided into two main groups: true and false aneurysms. While true aneurysms have a wide mouth and the wall is comprised of infracted/fibrous tissue [1], false aneurysms represent complete ruptures of the myocardial wall. They have a narrow neck and are contained by pericardium. In contrast to true aneurysms, false aneurysms have a dismal prognosis. Therefore, fast and accurate diagnosis and treatment can be life saving [2].

Impending wall ruptures and thus precursors to false aneurysms are called subepicardial aneurysms (SEA). They were first described by Hunter in 1933 as a rare form of saccular aneurysm [3]. In 1983 Epstein was first to use the term "subepicardial" aneurysms and described them as having three distinguishing features: abrupt interruption of the myocardium at the neck of the aneurysm, a narrow neck relative to the diameter of the aneurysm, and a propensity to rupture spontaneously [4]. SEAs are difficult to diagnose and are often only found post-mortem. In this case we report an impending rupture of an SEA in a patient with chest pain 4 days post AMI. This diagnosis was made using computed tomography (CT) and magnetic resonance imaging (MRI), which assisted in securing a favorable patient outcome.

## Case presentation

**Figure 1:**
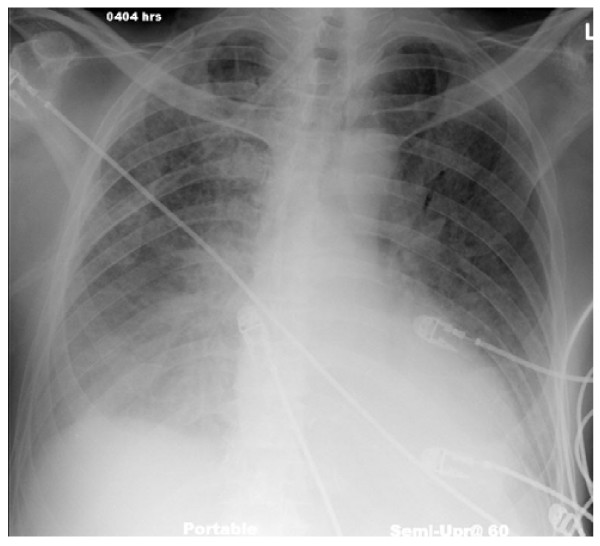
**Portable AP chest radiograph of a 67 year old patient with pulmonary edema, small bilateral pleural effusions, and cardiomegaly five days post myocardial infarction**.

A 67 year-old patient presented to the emergency room with worsening chest pain and shortness of breath four days post acute inferolateral myocardial infarction with subsequent left circumflex coronary artery stent placement. The patient had no history of prior myocardial infarctions. The chest radiograph showed moderate pulmonary edema and small bilateral pleural effusions (Fig. 1).

**Figure 2:**
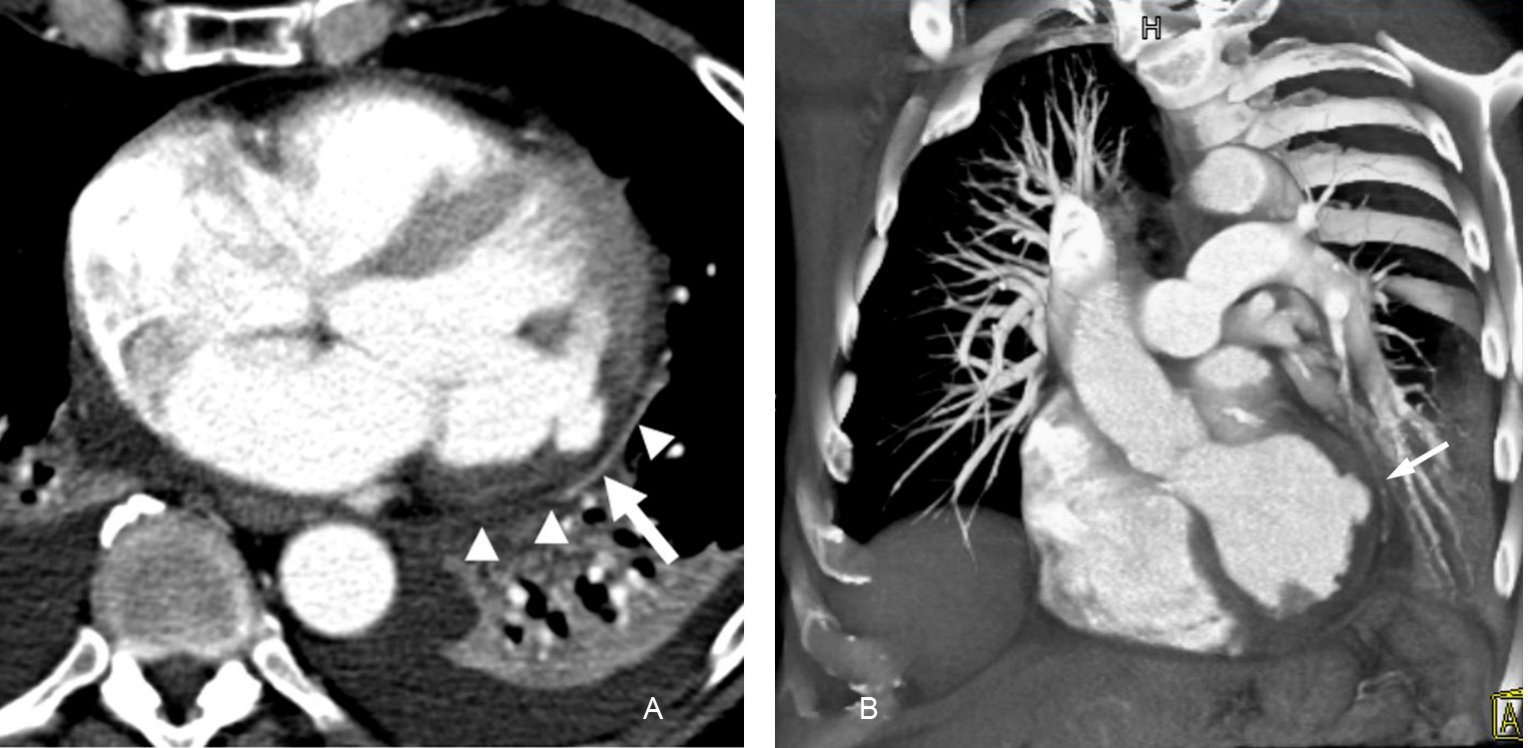
**Axial contrast enhanced CT image of the chest (a) shows an area of decreased perfusion in the lateral wall of the left ventricle (arrowheads) with a 1 × 1.6 cm blister-like pouch (arrow)**. A volume rendered 3D MDCT image (b) of the left ventricle shows an area of localized contrast out-pouching with a narrow neck in the lateral left ventricular wall (arrow).

To rule out pulmonary embolism, the patient was referred for a computed tomography (CT) scan. The CT was negative for pulmonary embolus; however, an incidental 1.0 × 1.6 cm blister-like pouch/aneurysm was seen in the lateral LV wall within a hypoperfused area that extended from the lateral to the inferoseptal wall and from the base to the mid-cardiac level (Fig. 2). This finding was concerning for an SEA/impending myocardial rupture within the subacutely infarcted left ventricular wall. However, it was deemed necessary to further characterize the anatomy of the infarct and myocardial outpouching to determine the urgency for cardiac surgery.

**Figure 3:**
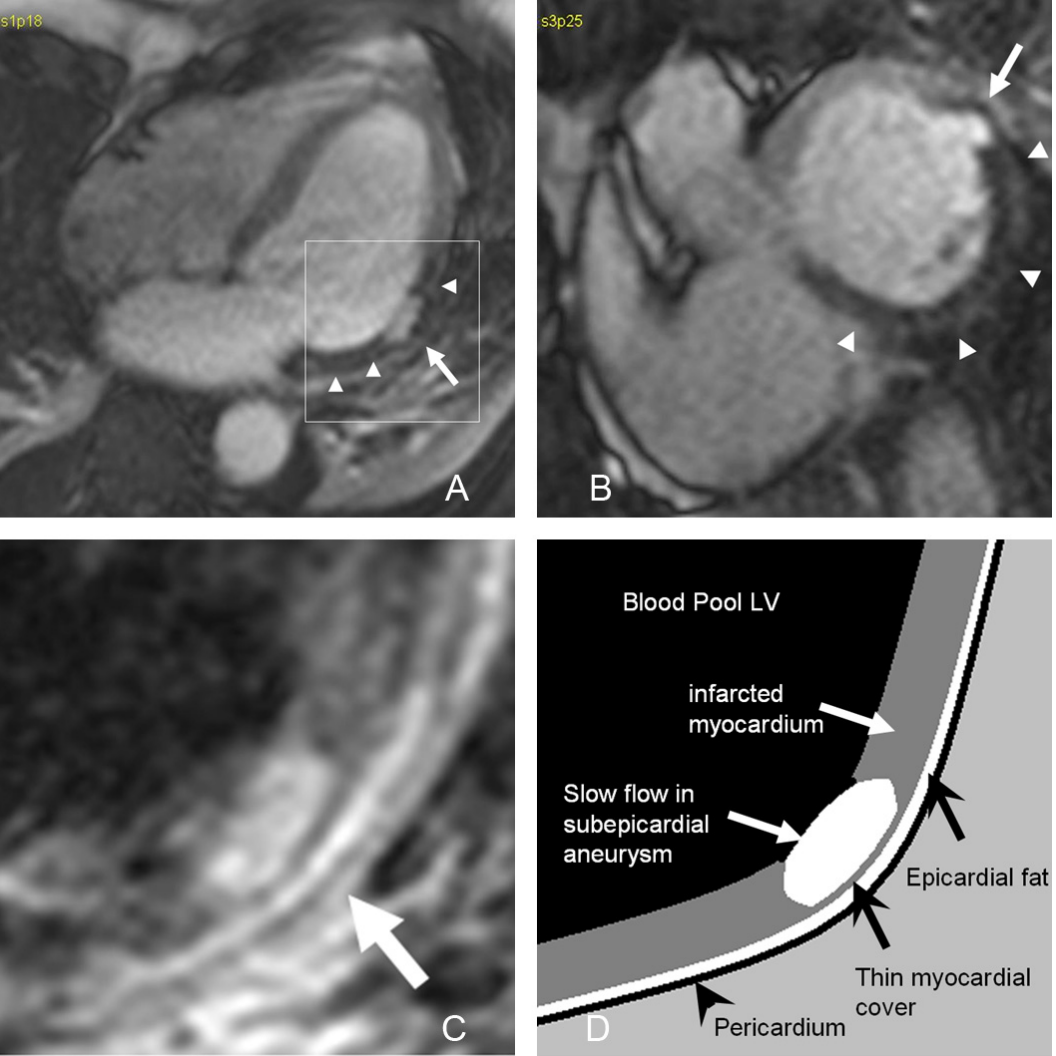
**Axial (a) and short axis (b) first pass perfusion SSFP MR images demonstrate a large area of microvascular obstruction in the inferolateral and inferoseptal left ventricular wall (arrowheads) with an area of blister-like contrast pouch covered by a 1 mm thin rim of infarcted myocardial tissue (arrow) compatible with an impending left ventricular rupture**. The magnified view (c, the area is indicated by the square in Fig. 3a) of an axial T1 weighted double inversion FSE MR image confirms the thin myocardial cover (arrow) of this subepicardial aneurysm (arrow), which has bright signal due to slower flow compared to the left ventricular blood pool. The overlying epicardial fat (arrowhead) and pericardium are normal. Figure 3d represents a drawing of the complex anatomy in figure 3c.

**Figure 4:**
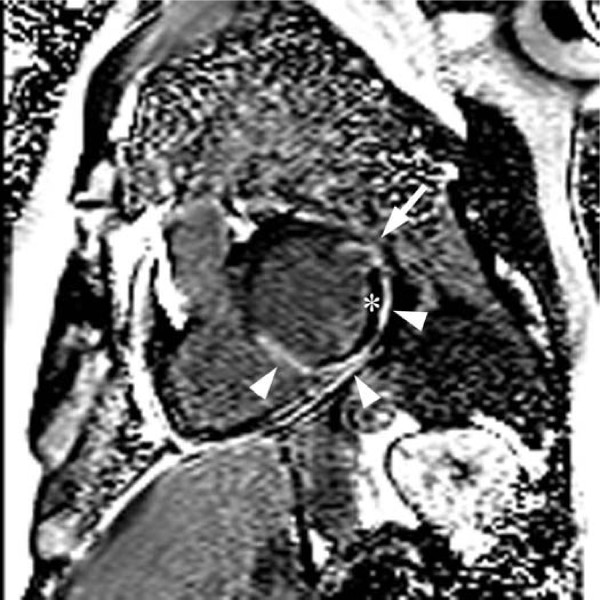
Short axis delayed enhancement inversion recovery MR image with phase correction after 55 minutes (SSFP-GRE) post intravenous gadolinium injection shows the large inferolateral and inferoseptal acute myocardial infarction (arrowheads) with a persistent large area of microvascular obstruction (*). The impending rupture site in the lateral left ventricular wall shows delayed enhancement of the thin overlying cover of infracted myocardium (arrow).

Because the patient was hemodynamically stable, cardiac magnetic resonance (CMR) imaging was performed. Cardiac MR images demonstrated a large subacute inferoseptal, inferior, and lateral transmural myocardial infarction with extensive microvascular obstruction on the first pass perfusion images (Fig. 3a and 3b, see additional files 1 and 2). The first pass perfusion defect persisted on the delayed enhancement MR images taken 55 minutes after the gadolinium injection (Fig. 4). At the lateral edge of the infarction, a small aneurysm with a narrow neck was identified (see additional file 3), consistent with the CT findings. The aneurysm was covered by only 1 mm of infarcted myocardium (Fig. 3c). There was no evidence of rupture into the pericardium.

During surgery, which was performed within 24 hours of CT/MR imaging, a distinct area of thin and weak myocardium in the lateral left ventricular wall was evident. The epicardium was intact and the area correlated with the preoperative imaging. Since the region was very close to the base of the heart as well as the AV groove, a bovine pericardial patch was sewn over the region using a continuous prolene suture. The patch was reinforced with a thin layer of Bioglue^® ^adhesive (Cryolife, Inc). At the same time, coronary bypass grafting and a mitral valve repair were performed to treat the patient's ischemic heart disease and severe mitral valve insufficiency. The patient was discharged home on post operative day eight in good condition and is feeling well 2 years after surgery.

## Discussion

After an acute myocardial infarction (AMI), there are several potentially life-threatening complications: (1) Arrhythmias [5], (2) cardiogenic shock, (3) complete free wall ruptures which account for almost 4% of patients' deaths after AMI (33% occur within the first 24 hours, 85% within the first week [6]), (4) complete septal ruptures (accounting for 1% – 5% of all infarct-related deaths [7]), and (5) the formation of false aneurysms.

While true aneurysms typically do not require treatment, false aneurysms, or pseudoaneurysms, are the result of a complete rupture of the ventricular wall with containment of the resulting hematoma by adherent pericardium and thus have a high mortality rate. As SEAs are precursors to pseudoaneurysms with a high propensity to rupture, immediate treatment is often life-saving. Although conservative management has been reported to be successful in asymptomatic chronic SEAs [8-10], surgical treatment is still considered standard of care, especially for symptomatic acute SEAs, as in our case [9,11-13]. The options include aneurysmectomy (resection) or aneurysmorrhaphy (patch repair) [11]. In addition to an elevated risk of death, patients with SEAs are initially difficult to diagnose due to a lack of specific symptoms (our patient was suspected to have a pulmonary embolus). Diagnosis can be made using ultrasound, MRI, left heart catheter, or CT [11]. Due to the high risk of rupture in combination with the difficult diagnosis, SAEs have a high mortality rate and diagnosis is often made post-mortem.

SEAs are rare; in 1,814 autopsied hearts with 1,140 MIs (in 704 hearts), only three SEAs were found (0.2% of infarcts) [4]. Review of literature reveled 36 published cases to date. As in our case, SEAs typically occur post AMI, usually within the first few weeks. Additionally, there are reports of SEAs (1) in an avascular region without history of AMI or signs of coronary artery disease [14], (2) as a direct result of apicoaortic bypass [15], and (3) after repair of a ventricular septal rupture [16].

## Conclusion

In a patient with continued chest pain post-AMI, subendocardial left ventricular aneurysm/impending rupture should be considered as an uncommon yet life-threatening differential diagnosis. In this case, the SEA was visible on the pulmonary embolism CT scan as an incidental finding and confirmed on a dedicated cardiac MRI. Emergency surgery guided by these imaging findings most likely saved the patient's life.

## Consent

Written informed consent was obtained from the patient for publication of this case report and any accompanying images. A copy of the written consent is available for review by the Editor-in-Chief of this journal.

## Competing interests

The authors declare that they have no competing interests.

J.V.C. was supported by the Radiological Society of North America Research and Education Foundation.

## Authors' contributions

JVC conducted the CT and MRI exams and drafted the manuscript. JS drafted the manuscript and conducted the literature search. ASS was directly involved in the patient care. EKF, JACL, ASS and DAB substantially revised and edited this manuscript.

## Supplementary Material

Additional file 1 **First pass resting perfusion short axis MRI**. Extensive microvascular obstruction in the inferoseptal and inferolateral left ventricular wall. Click here for file

Additional file 2 **First pass resting perfusion long axis MRI**. Extensive microvascular obstruction in the lateral left ventricular wall. Click here for file

Additional file 3 **Cine short axis MRI**. Akinesia at the inferior wall and moderate hypokinesia at the inferior septum and lateral wall with the focal impending left ventricular rupture in the lateral wall. Click here for file
